# Role of stress-related glucocorticoid changes in astrocyte-oligodendrocyte interactions that regulate myelin production and maintenance

**DOI:** 10.14670/HH-18-476

**Published:** 2022-06-02

**Authors:** José Javier Miguel-Hidalgo

**Affiliations:** University of Mississippi Medical Center, Department of Psychiatry and Human Behavior, Jackson, MS, USA

**Keywords:** Corticosteroids, Connexin, Glia, Myelination, Chronic stress, Animal model

## Abstract

Repeated activation of stress responses and elevated corticosteroids result in alterations of neuronal physiology and metabolism, and lead to disturbances of normal connectivity between neurons in various brain regions. In addition, stress responses are also associated with anomalies in the function of glial cells, particularly astrocytes and oligodendrocytes, which in turn may further contribute to the mechanisms of neuronal dysfunction. The actions of corticosteroids on astrocytes are very likely mediated by the presence of intracellular and cell membrane-bound CORT receptors. Although apparently less abundant than in astrocytes, activation of CORT receptors in oligodendrocytes also leads to structural changes that are reflected in myelin maintenance and plasticity. The close interactions between astrocytes and oligodendrocytes through extracellular matrix molecules, soluble factors and astrocyte-oligodendrocyte gap junctions very likely mediate part of the disturbances in myelin structure, leading to plastic myelin adaptations or pathological myelin disruptions that may significantly influence brain connectivity. Likewise, the intimate association of the tips of some astrocytes processes with a majority of nodes of Ranvier in the white matter suggest that stress and overexposure to corticosteroids may lead to remodeling of node of Ranvier and their specific extracellular milieu.

## Introduction

Significant exposure to the stress response results in profound functional and metabolic alterations in specific brain areas (Nitschke et al., 2000; McEwen, 2007; Pruessner et al., 2010; Radahmadi et al., 2017; van der Kooij, 2020). Some of these regions, such as the anterior cingulate cortex, amygdala and hippocampus, are differentially affected by stress modalities like those causing early life stress, repeated daily stresses, bereavement, or loss of social status (Kaul et al., 2021). Eventually, disturbances in these brain areas are reflected in functional disruption of brain networks involved in establishing the salience of environmental information, supporting cognitive and emotional processing or setting the activity of default mode brain networks (Koch et al., 2016; Ebmeier and Zsoldos, 2019). Since drastically increased levels of glucocorticoids (CORTS) is a major feature of the immediate response to stress, human studies have also targeted the in vivo correlation of specific brain activity/metabolic changes with CORTS increases and their time course (Madalena and Lerch, 2017). Other research in human subjects has further shown the ability of experimentally elevated CORTS to elicit structural or functional brain changes in vivo (Lovallo et al., 2010; Schwabe et al., 2012; Strelzyk et al., 2012; Szeszko et al., 2018), while work on postmortem human brain samples has examined cellular and neurochemical alterations, such as glucocorticoid receptor abnormalities, associated with stress and CORT elevation (Swaab et al., 2005; Wolf, 2006; Wang et al., 2014). Furthermore, various animal models for the induction of stress responses by physical or social procedures or by exogenously elevating systemic CORTS, have also demonstrated alterations in brain activity and metabolism comparable to those detected in humans under stress or elevated glucocorticoids (Kaul et al., 2021). In addition, animal in vivo research and in vitro models have demonstrated cellular and molecular mechanisms responsible for functional and structural alterations at the level of synapses, individual neurons or defined neuronal populations resulting from exposure to high glucocorticoid levels (Yuen et al., 2012; Treccani et al., 2014; McEwen et al., 2016; Qiao et al., 2016). More recently, given the critical role of glial cells and the regulation of neuronal activity and survival, the scope of stress research has significantly expanded to include the involvement of glial cells, such astrocytes and oligodendrocytes in the pathophysiology of stress responses and the alterations in brain circuits underlying the behavioral and neurological consequences of stress and excess CORTS (Edgar and Sibille, 2012; Madalena and Lerch, 2017; Leng et al., 2018; Cathomas et al., 2019; Murphy-Royal et al., 2019), although the importance of glial involvement and its possible mechanisms has remained less studied overall. The present review concentrates on the effects of stress and glucocorticoids on the biology of astrocytes and oligodendrocytes as well as on the possibility that interactions between astrocytes and oligodendrocytes represent a mechanism by which glial cells contribute to functional alterations caused by excess glucocorticoid exposure.

## Astrocytes in stress

In animal models of stress, and in psychiatric disorders with stress as a main risk factor, research has revealed that astrocytes from various brain regions display significantly altered morphology and neurochemistry as compared to non-stressed controls or non-psychiatric subjects (Bender et al., 2016; Murphy-Royal et al., 2019). Different modalities of chronic stress based on restraint, unpredictable stressors or social defeat in rodents have shown that astrocyte numbers labeled with specific markers such as GFAP, and the extent of the GFAP-positive processes stemming from astrocyte cell bodies, are reduced in various brain regions including the prefrontal cortex and the hippocampus and that there is a reduction also in the corresponding GFAP mRNA (Banasr and Duman, 2008; Liu et al., 2009; Banasr et al., 2010; Araya-Callis et al., 2012; Imbe et al., 2012, 2013; Li et al., 2013; Tynan et al., 2013). Similar results have been obtained by studying a rat strain, the Wistar Kyoto (WK) rats, that are more prone to show depression- and anxiety-like behaviors than other strains (Gosselin et al., 2009). WK rats also demonstrate lower GFAP immunostaining and levels in cortical and subcortical brain regions. In tree shrew, a marsupial mammal, the numbers and cell body size of GFAP immunoreactive astrocytes are also smaller in the hippocampus after stress caused by social defeat (Czeh et al., 2006).

A major feature of astrocyte biology is the establishment of gap junctions between astrocytes or between astrocytes and oligodendrocytes to allow for direct diffusion-based, intercytoplasmic exchanges of small metabolites and ions. These junctions are formed by aggregates of channels directly putting in contact the cytoplasms of contiguous cells. Each side of the channel is composed of cell type-specific proteins called connexins, six of these subunits arrange around a channel (called connexon) that is contiguous with the connexon formed by another six subunits in the membrane of the adjacent cell. The connexin subunits of astrocytes that form their side of a majority of their gap junctions are connexin 43 (Cx43) or connexin 30 (Cx30). Chronic unpredictable stress (CUS) in rats was found to reduce gap junction function, the Cx43 immunoreactivity of gap junction aggregates and led to dramatic reductions in the expression of Cx43 and its mRNA that were preventable by treatment with glucocorticoid receptor antagonist mifepristone (Sun et al., 2012; Miguel-Hidalgo et al., 2019), also preventing the expression of depression-like behaviors. More recently, we observed that the density of Cx43 and Cx30 immunoreactive aggregates in histological sections from the rat prefrontal cortex were depleted in CUS rats as compared to non-stressed rats (Miguel-Hidalgo et al., 2018). In the same brain region immunostaining for myelin basic protein (MBP) was also significantly reduced in CUS rats (Miguel-Hidalgo et al., 2018) as compared to controls.

## Oligodentrocytes in stress

The stress response has been proposed to aggravate myelin-related pathologies. At least part of the stress effects on myelin formation and maintenance may be mediated by alterations of oligodendrocyte physiology, metabolism and gene expression. For instance, in the adult mouse the transcription of mRNAs for myelin related proteins was significantly altered by CUS or social stress (Laine et al., 2018; Liu et al., 2018; Cathomas et al., 2019). Interestingly myelin and oligodendrocyte-related transcripts were decreased in the prefrontal cortex and nucleus accumbens but increased in the corpus callosum (Liu et al., 2018), suggesting a brain-region specific pathology. Also in the prefrontal cortex (but not in the cerebellum) the thickness of myelin sheaths, as visualized by electron microscopy, was found to be reduced in mice subjected to several weeks of social isolation, and this change was partially reversible after re-socialization (Liu et al., 2012). Another study in CUS mice revealed reductions in oligodendrocyte differentiation and the expression of myelin proteins in the medial PFC that could be reversed by an exercise regime, which has antidepressant-like effects (Luo et al., 2019). In a model of restraint stress, forebrain myelin thickness was also smaller in restrained animals and was reversible by exposure to an enriched environment (Thamizhoviya and Vanisree, 2019). Interestingly, in the medial PFC, but not in the hippocampus, of adult mice exposed to social defeat stress during adolescence there was a decrease in myelin basic protein, although the numbers of oligodendrocytes did not change (Xu et al., 2020). In the gray matter, stress-related myelin alterations might be sex-dependent since exposure to traumatic stress in juvenile rats results in disturbances of myelination in adult female but not male rats (Breton et al., 2021). Unlike chronic forms of stress, acute exposure to a severe stressor in male rats induced avoidance behaviors that were positively correlated with increased myelin proteins in the dentate gyrus of hippocampus, while contextual fear learning was associated with increased myelin in the amygdala and spatial processing portions of the hippocampus (Long et al., 2021). On the other hand, induced-overexpression of oligodendrocyte transcription factor Olig1 in the dentate gyrus enhanced fear- related and anxiety-like responses. Interestingly, in human subjects severity of PTSD symptoms was also found to be positively correlated with neuroimaging-based estimates of myelin content in the amygdala and hippocampus, but not in the corpus callosum (Long et al., 2021). The studies above thus suggest not only that stress affects regional myelin parameters probably depending on whether it is acute or chronic, but also that vulnerability to stress-related fear/anxiety behaviors may depend on the involvement of oligodendrocytes and myelin in the responses to stress (Long et al., 2021).

### Glucocorticoids and astrocytes

Astrocytes in both the white and gray matter have been found to carry glucocorticoid receptors (Vielkind et al., 1990; Bohn et al., 1991; Vardimon et al., 1999) and the activation of those receptors causes significant alterations of calcium transients and morphological features in astrocytes in vitro and in vivo (Simard et al., 1999; Zeng et al., 2013). Acute exposure of cultured cortical astrocytes results in the cell-specific and non-specific regulation of mRNAs for many proteins following a particular time course after exposure (Carter et al., 2012, 2013). Some research has been dedicated to the effects of astrocyte GR activation by corticosterone on the expression of cytoskeletal protein GFAP, showing that GFAP is transcriptionally regulated by CORT in vitro and influenced by coculture with neurons (Rozovsky et al., 1995; Joshi and Benerjee, 2018). In vivo, CORT also regulates the numbers of GFAP positive brain expression in the hippocampus (Bridges et al., 2008; Lou et al., 2018) and these effects on astrocytes and behaviors were curtailed by infusion of the GR antagonist mifepristone (Lou et al., 2018). Prolonged treatment with glucocorticoids in vivo produces a decrease in GFAP protein and mRNA expression in cortex and other various brain regions in rats (O’Callaghan et al., 1991). In addition, chronic administration of corticosterone in mice induces a reduction in the number of GFAP-immunoreactive astrocytes, the volume of their cell bodies, and the length of their processes in the hippocampus (Zhang et al., 2015). In vitro, high levels of CORT or GR agonist in mixed cultures (neurons, astrocytes and oligodendrocytes) appear not to reduce the numbers of astrocytes or GFAP staining but their expression of connexin 43, a gap junction protein crucial for astrocyte-to-astrocyte communication, is dramatically reduced following repeated application of high glucocorticoid levels. Astrocytes both in the gray and white matter express glutamate transporters and glutamine synthetase which are essential for the reuptake and recycling of released glutamate. In WM glutamate is linked to the activation of myelination. Glutamine synthetase in astrocytes is regulated by corticosteroids (Vardimon et al., 1999) suggesting a pathway for the actions of CORT on myelination through activation of astrocytic GRs.

There has been significant research on the specific pathways activated by GRs in astrocytes that would be involved in the pathological consequences of stress as a major risk factor for depression and other psychiatric disorders. For instance, astrocyte-specific depletion of GRs results in cognitive and behavioral deficits that have been associated with the stress response, such as reduced expression of aversive memories (Tertil et al., 2018). These effects were mediated by the dysregulation of stress-activated molecule Sgk1 which participates in the regulation of glucose metabolism. Lactate production is a major process depending on glucose metabolism in astrocytes that has been shown to depend also on glucocorticoid activation. in the nucleus accumbens this lactate regulation appears to be a major determinant for conditioned responses to morphine (Skupio et al., 2020). On the other hand, activation of corticosteroid receptors of astrocytes leads to the release of neuroinflammatory mediators such as high-mobility group box-1 (HMGB1), of importance because neuroinflammation is considered a mechanism contributing to depression-like behaviors (Hisaoka-Nakashima et al., 2020). This role of astrocyte glucocorticoid receptor activation in neuroinflammatory regulation is also supported by the induction of lipocortin-1 in cultured rat astrocytes exposed to corticosteroid analog dexamethasone (McLeod and Bolton, 1995).

### Glucocorticoids, oligodendrocytes and myelin

Oligodendrocytes and oligodendrocyte precursor cells (OLP, also called NG2 cells) express glucocorticoid receptors although their levels appear to be lower than in astrocytes (Vielkind et al., 1990; Matsusue et al., 2014). Some of the available studies on the effects of increased glucocorticoids or their analogs on oligodendrocytes and the myelin they form have found that glucocorticoids play an inhibitory role in myelin formation as observed in both in vivo and in vitro experimental systems (Meyer, 1983; Antonow-Schlorke et al., 2009; Jauregui-Huerta et al., 2010). That role extends to oligodendrocyte precursors cells (OLP) which are prevented from differentiating into full-fledged oligodendrocytes by the prolonged exposure to glucocorticoids in both the brain and spinal cord (Alonso, 2000; Schroter et al., 2009). However, it seems that at particular stages of development glucocorticoids stimulate or favor the expression of myelin components such as MBP, PLP or GAPDH in oligodendrocytes by different transcriptional mechanisms (Kumar et al., 1989). In disorders of myelin such as MS, glucocorticoids are indicated to slow demyelination, although it is unclear whether the effects are mediated by GRs in oligodendrocytes or are rather an indirect consequence of GR activation in other cells or organs (Zivadinov et al., 2001). On the other hand, positive effects of corticosteroids on the initiation of myelination or oligodendrocyte survival have been shown in cell cultures after excitotoxicity or cell death induction, or in vivo after spinal cord injury and at least some of these effects depend on the activation of GRs (Lee et al., 2008; Xu et al., 2009; Sun et al., 2010), while in neuron/Schwann cell cocultures GR activation seems to accelerate the initiation and rate of myelination (Chan et al., 1998). Besides the effects of oligodendrocyte GRs on myelination GR activation in oligodendrocytes also regulates the expression of metabolic and survival transcription factor Sgk-1 indicating a significant involvement of oligodendrocyte plasticity in the responses to stress (Hinds et al., 2017).

## Glucocorticoids in the interaction between astrocytes and oligodendrocytes

In addition to the effects that corticosteroids may separately exert on astrocytes and oligodendrocytes, effects of elevated glucocorticoids on myelination and oligodendrocyte differentiation/proliferation may likely depend on disturbances of the interactions between astrocytes and oligodendrocytes (Nutma et al., 2020; Tognatta et al., 2020), particularly those based on their communication through gap junctions. In addition to homologous astrocyte-to-astrocyte gap junctions (supported by connexins 43 and 30), astrocyte-to-oligodendrocyte heterologous gap junctions are ubiquitous in the white matter. These junctions are comprised of aggregates of astrocyte connexons (made each one of six Cx43 or Cx30 subunits lining a pore across the cell membrane) interacting with the corresponding connexons from oligodendrocytes (made each of 6 Cx47 or C32 subunits at the oligodendrocyte membrane) (Giaume and McCarthy, 1996). Opposing connexons from adjacent cells are in direct contact and define a channel for intercytoplasmic communication between astrocytes and oligodendrocytes. Accumulating evidence has demonstrated that myelin formation and stability is crucially dependent on those heterologous gap junctions since mice with KO of one or more of the subunits forming astrocyte-to-oligodendrocyte junctions display various levels of myelin vacuolization and disruption as well as grave behavioral abnormalities (Lutz et al., 2009; Magnotti et al., 2011; Li et al., 2014; Basu and Sarma, 2018). It is still unclear whether prolonged systemic alterations of glucocorticoids modify myelin synthesis or repair through disturbances in astrocyte-to-oligodendrocyte junctions. However, we have recently produced evidence that stress and high glucocorticoid concentrations lead to reduction both in immunohistochemical labeling of myelin markers and in the labeling of Cx43-positive aggregates in two different subdivisions of the prefrontal cortex (Miguel-Hidalgo et al., 2018). The magnitude of Cx43 and myelin changes detected associated with stress responses does not seem to be as large as that in connexin KO animals. Nevertheless, mounting research suggests that the structure of myelin is susceptible to undergo plastic changes within physiological parameters that result in meaningful variations of neural function and brain connectivity (Nave, 2010; Nave and Ehrenreich, 2014; Fields, 2015; Fields et al., 2015).

In addition, in cell culture experiments with a primary mixed population of neural cells (astrocytes, oligodendrocytes and neurons) from the rat frontal cortex, myelination was dramatically diminished by the repeated exposure to high corticosterone (Miguel-Hidalgo et al., 2019). In the same conditions, there was a concomitant depletion of astrocytic Cx43 immuno-histochemically labeled puncta, suggesting that both defective gap junction communication and myelin formation ensue the exposure to repeated excessive corticosterone. Effects both on myelin and on gap junctions were effectively prevented by the application of mifepristone, a potent antagonist of glucocorticoid receptors (Miguel-Hidalgo et al., 2019).

Astrocytes, although not exclusively, are also main contributors to extracellular matrix components and their regulation in gray and white matter that surround the initial segments (AISs) of myelinated axons (where action potentials are generated) and the nodes or Ranvier (NRs) (where action potentials are regenerated as they are carried to synaptic terminals) (Dutta et al., 2018; Rasband and Peles, 2021). Proteoglycans such as Bral1, versican, brevican, phosphacan and other proteins are produced abundantly by astrocytes and they associate with other ECM proteins, such as Tenascin R, eventually binding various motifs in axonal membrane cell adhesion proteins at NRs and AISs to stabilize and help with the aggregation of voltage gated sodium channels in the axonal membrane (Bekku and Oohashi, 2019; Rasband and Peles, 2021). In recent experiments on postmortem brain white matter underlying the orbitofrontal cortex of human subjects we have generated preliminary evidence of a significant increase in the levels of the fluorescent immunostaining for the proteoglycan phosphacan at NRs and phosphacan levels that are concomitant with a reduction in the length of NRs in subjects with depression as compared to non-psychiatric controls (Miguel-Hidalgo et al., 2021), suggesting that ECM components at NRs associated with astrocytes may be altered in subjects with depression and that those alterations may be related to structural remodeling of myelin segments adjacent to NRs, although further research is clearly required to fully ascertain the extent of those alterations and their putative influence on physiological processes involved in connectivity changes.

It has long been known that growth factors, including neurotrophins, secreted by astrocytes have an important role in regulating the myelination and remyelination processes (Moore et al., 2011; Barnett and Linington, 2013; Kiray et al., 2016; Molina-Gonzalez and Miron, 2019). Given that corticosteroids are capable of regulating the release of astrocyte-derived growth factors (Niu et al., 1997; Anacker et al., 2013; Karki et al., 2017), which in turn may modulate myelin formation (Barateiro and Fernandes, 2014; Langhnoja et al., 2021), growth factor-based regulation represents an additional mechanism by which astrocyte responses to a stress-induced CORTS increase can affect myelin plasticity.

## Conclusion

**Fig. 1.**
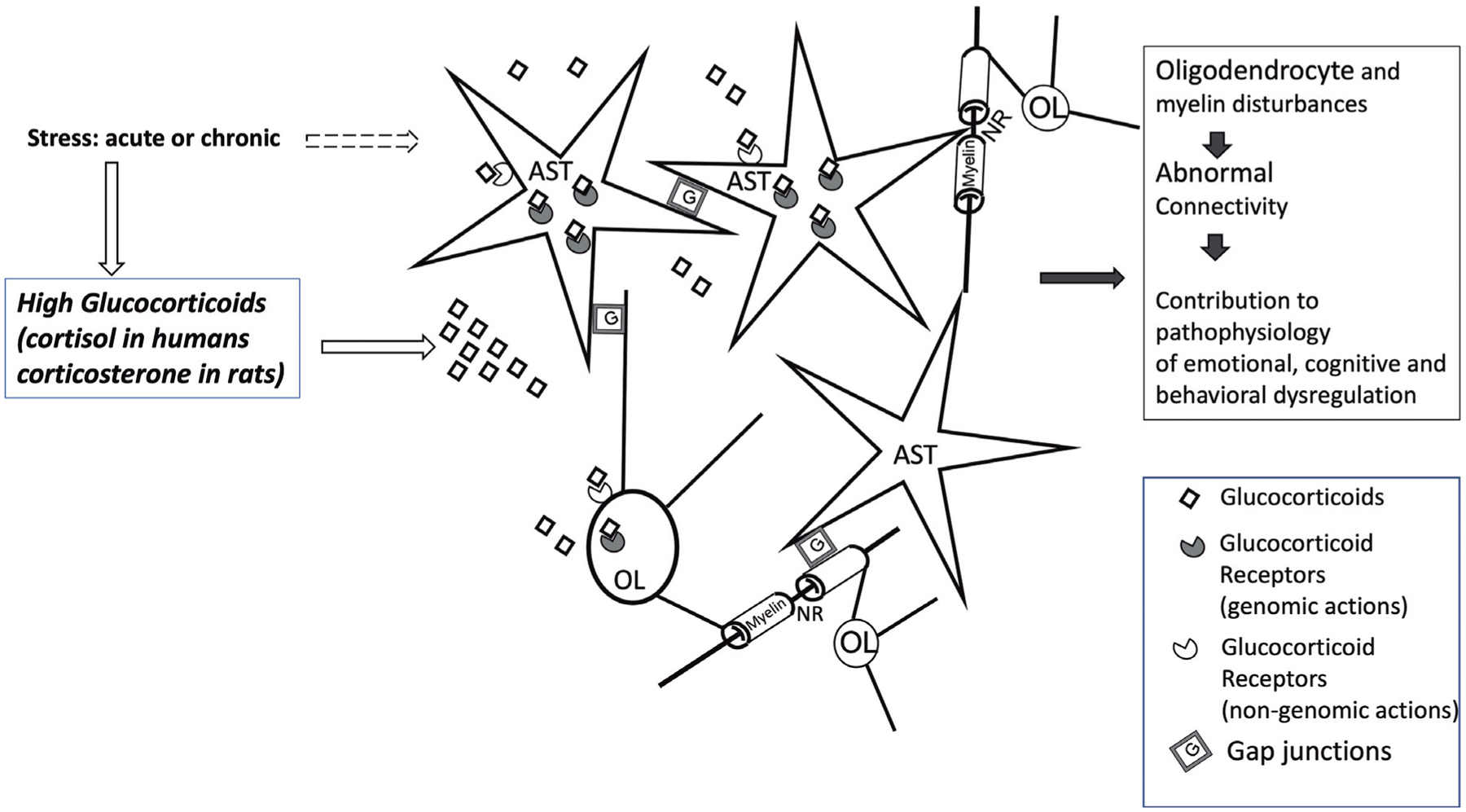
Illustration of relevant interactions involving CNS astrocytes and oligodendrocytes that would be affected by stress and the dramatic elevations of glucocorticoids during the stress response. The pathological activation of astrocytes and oligodendrocytes would result in a variety of molecular and neurotransmitters alterations in the gray matter and in the white matter connecting prefrontal cortex, hippocampus, amygdala, striatum, hypothalamus and other regions. Those alterations lead to dysregulation of connectivity due to the importance of signal propagation along myelinated axons connecting those brain regions. More recent research is revealing that regulation of nodes or Ranvier by astrocytes processes and oligodendrocyte paranodal regions is critically involved in shaping the propagation of action potentials. Nonetheless, a great deal of additional research remains to be done to significantly improve our understanding of the mechanisms that result in dysfunctional interactions between astrocytes, oligodendrocytes, and the neurons they serve, and the role of corticosteroid elevations in those mechanisms. The broken hollow arrow represents stress-related factors other than glucocorticoids that, while less-well understood, may modulate the interactions between astrocytes and oligodendrocytes. AST, Astrocyte; G, Gap junctions; NR, Node of Ranvier; OL, Oligodendrocyte.

The association of astrocytes and oligodendrocytes through gap junction communication, extracellular matrix components and trophic factors, and the ability of those two cell types to respond to glucocorticoids through glucocorticoid receptors suggest that the interactions between those glial cells may play a important role in the neural mechanisms of stress responses (Fig. 1). Some of these interactions may be reflected in the reorganization of myelin sheaths or the plasticity of de novo myelin production, while other glucocorticoid-driven changes may affect the morphology of NRs or fundamental NR properties relevant to action potential generation such as the aggregation of voltage-gated sodium channels and ion diffusion regulation.
